# Psychological distress, self-harm and suicide attempts in gender minority compared with cisgender adolescents in the UK

**DOI:** 10.1192/bjo.2023.534

**Published:** 2023-08-01

**Authors:** James White, Mai-Han Trinh, Colleen A. Reynolds

**Affiliations:** Centre for Trials Research, School of Medicine, Cardiff University, Cardiff, UK; and DECIPHer, School of Social Sciences, Cardiff University, Cardiff, UK; Department of Epidemiology, Harvard T. H. Chan School of Public Health, Boston, USA

**Keywords:** Gender identity, psychological distress, self-harm, suicide attempt

## Abstract

**Background:**

Few population-based studies have compared the mental health of gender minority and cisgender adolescents.

**Aims:**

To compare reports of psychological distress, behavioural and emotional difficulties, self-harm and suicide attempts between gender minority and cisgender adolescents.

**Method:**

Data came from the Millennium Cohort Study (*n* = 10 247), a large nationally representative birth cohort in the UK. At a 17-year follow-up, we assessed gender identity, psychological distress (Kessler K6 scale), behavioural and emotional difficulties (parent and child reports on the Strengths and Difficulties Questionnaire), self-harm in the previous year, suicide attempts, substance use, and victimisation including harassment and physical and sexual assaults. Multivariable modified Poisson and linear regression models were used. Attenuation after the inclusion of victimisation and substance use was used to explore mediation.

**Results:**

Of the 10 247 participants, 113 (1.1%) reported that they were a gender minority. Gender minority participants reported more psychological distress (coefficient 5.81, 95% CI 4.87–6.74), behavioural and emotional difficulties (child report: coefficient 5.60; 95% CI 4.54–6.67; parent/carer report: coefficient 2.60; 95% CI 1.47–3.73), self-harm including cutting or stabbing (relative risk (RR) 4.38; 95% CI 3.55–5.40), burning (RR 3.81; 95% CI 2.49–5.82), taking an overdose (RR 5.25; 95% CI 3.35–8.23) and suicide attempts (RR 3.42; 95% CI 2.45–4.78) than cisgender youth. These associations were partially explained by differences in exposure to victimisation.

**Conclusions:**

Gender minority adolescents experience a disproportionate burden of mental health problems. Policies are needed to reduce victimisation and services should be adapted to better support the mental health of gender minority adolescents.

Gender minority adults (those with an identity that differs from their assigned sex at birth) are more likely than cisgender adults (those with an identity that corresponds to sex assigned at birth) to report symptoms of depression and anxiety and to self-harm and attempt suicide.^1,2^ The few studies on the mental health of gender minority adolescents have used convenience sampling, which might introduce sampling bias; have been small and so might lack statistical power; or did not match cisgender comparators, preventing the estimation of differences by gender identity.^3–5^ In the one nationally representative study in New Zealand, symptoms of depression, anxiety, self-harm and suicide attempts were elevated in gender minority compared with cisgender adolescents;^6^ however, the study did not examine the role of substance use or victimisation in these associations. Two US studies^4,7^ found that transgender young people reported more smoking, alcohol and illicit drug use as well as more victimisation than their cisgender peers, suggesting that these variables may have a role in the association between gender minority status and mental health.

The UK's Millennium Cohort Study (MCS), with its assessments of psychological distress, emotional and behavioural difficulties, self-harm and suicide attempts, as well as reports of substance use and victimisation ranging from insults to sexual assaults, in a nationally representative sample represents a unique opportunity to explore the association between gender identity and mental health.

## Method

### Setting and participants

The MCS is a birth cohort in the UK following children born in 2000–2002. In total, 19 519 children were recruited and have been followed up seven times to date at ages 9 months and 3, 5, 7, 11, 14 and 17 years. For information regarding the design of the MCS, see https://cls.ucl.ac.uk/cls-studies/millennium-cohort-study/.

We used data gathered at 9 months and 3 years of age on assigned sex. The outcomes and covariates in the analysis were assessed at 17 years of age (2018–2019), except ethnicity, which was only reported by young people at 14 years of age. In the sweep when cohort members were 17 years of age, 14 496 families were invited to participate. Of this number, 10 625 (73.2%) families and 10 345 (71.4%) adolescents provided informed written consent and were interviewed.

Ethics approval for the age-17 sweep was obtained from the National Research Ethics Service Research Ethics Committee North East – York (ref: 17/NE/0341). Collected data are anonymised and available to researchers via the UK Data Service. We adhered to the Strengthening the Reporting of Observational studies in Epidemiology guidelines in this work.^8^

### Measures

#### Mental health outcomes

Participants responded to the validated K6 measure of psychological distress.^9^ This measure asks respondents how often in the past 30 days they felt, for instance, worthless, with five response options ranging from none to all of the time. Total scores range from 0 to 24, with higher scores indicating greater distress.

Parent/carers and young people completed the Strengths and Difficulties Questionnaire (SDQ),^10^ a validated screening tool to measure child and adolescent behavioural and emotional difficulties.^11^ The SDQ consists of four subscales that rate areas of behavioural and emotional difficulties (conduct problems, hyperactivity, emotional symptoms and peer problems), with each consisting of five items on a three-point scale. Individual item scores were summed to produce a continuous total score.

Self-harm was reported as a binary response (never harmed = 0; harmed = 1) to the question ‘During the last year, have you hurt yourself on purpose in any of the following ways?’, with separate questions for the methods of: cut or stabbed; burned, bruised or pinched; overdose; pulled out hair; and other. This question has not been validated, but our analyses focused on self-harm in the previous year as this is more clinically relevant and less prone to recall bias than self-harm occurring more than a year ago.^12^

Attempted suicide was reported with the question ‘Have you ever hurt yourself on purpose in an attempt to end your life?’. This question has not been validated. We derived a binary measure of lifetime suicide attempt from responses (never attempted suicide = 0; made a suicide attempt = 1).

#### Gender identity

Gender identity was assessed using self-reports from participants at 17 years of age with the question ‘Which of the following describes how you think of yourself?’ and the response options of: ‘male’, ‘female’ and ‘in another way’. Those selecting ‘in another way’ then provided a description that was coded into: ‘androgenous (male and female)’, ‘gender fluid’, ‘non-binary’ and ‘other.’ We also compared the gender identification provided by young people at 17 years with the sex provided by parent/carers when they were 9 months and 3 years of age. Parent/ carers at 9 months and 3 years could only report whether a child was male or female. We derived participants’ gender minority status using both parents’ and young people's responses. If young people at 17 years identified with a gender that was: ‘other’, ‘androgenous (male and female)’, ‘gender fluid’ or ‘non-binary’, they were categorised as a gender minority. If the sex reported by the parent/carer at 9 months or 3 years did not match that reported by young people at 17 years of age (e.g. the parent response at 9 months was male and the young person's response at 17 years was female), the participant was also categorised as gender minority.

Preliminary analysis (before imputation) categorised 109 (1.1%) participants as a gender minority. Of these, 58 (53.2%) were categorised in this way based on young people's self-reports. The remaining 51 were categorised by from comparing participants’ reported male or female gender identity at 17 years of age with the gender identity of the participant reported by the parent/carer at 9 months or 3 years of age.

#### Covariates

To describe the characteristics of gender minority young people compared with their cisgender peers, we analysed self-reported data collected on demographic characteristics including housing tenure (i.e. rented, owned), parent/carer composition in household (single parent or carer, or both parents or carers), responding parent/carer employment status, adolescent's ethnicity (i.e. White; ethnic minorities: mixed, Indian, Bangladeshi or Pakistani, Black or Black British, other ethnic groups) and sexual identity. Sexual identity was adjusted for given the link between gender and sexual identity and associations between sexual identity and mental health. Sexual identity was self-reported according to categories of completely heterosexual/straight, mainly heterosexual/straight, bisexual, mainly gay or lesbian, completely gay or lesbian, other, do not know and prefer not to say. In the unimputed data-set, 0.9% (*n* = 90) indicated they were mainly gay or lesbian, 1.6% (*n* = 160) completely gay or lesbian, 10.6% mainly heterosexual (*n* = 1101) and 6.3% (*n* = 656) bisexual. There is strong evidence that adolescents identifying as mainly heterosexual or not sure have an increased risk of mental health problems compared with those reporting they are completely heterosexual.^13,14^ To be consistent with this literature, participants reporting they were mainly heterosexual were categorised as bisexual, and those indicating that they were mainly or completely gay or lesbian were collapsed into one category. We assessed two hypothetical mediators of associations between gender minority status and mental health outcomes: substance use and victimisation. Substance use comprised lifetime smoking experimentation (including those who had only had one puff of a cigarette), consumption of a whole alcoholic drink and illicit drug use. Victimisation assessments were self-reports of experience over the past 12 months of nine forms of harassment, abuse and violence.

### Statistical analysis

A detailed description of attrition in the cohort has been provided elsewhere.^15^ Missing data per variable ranged from 2.3 to 12.9%. Participants who reported that they ‘do not know’, ‘prefer not to say’ or ‘do not want to provide’ their gender (*n* = 47), sexual identity (*n* = 51) or ethnic identity (*n* = 56) were removed from the sample. There were 7829 participants with no missing data on the variables used in our statistical models, making up the complete data sample. The imputed analytical sample had 10 247 participants. We assumed missingness was dependent on the observed data and imputed 20 data-sets by multiple imputation using chained equations. The imputation prediction model included all other analysis variables, along with combined sampling and attrition weights^16^ and an indictor variable denoting whether or not participants were the only cohort member in the household. Estimates were obtained by pooling results across 20 imputed data-sets, and the Monte Carlo errors suggested that this was a suitable number.^17^

The association between gender minority status and outcomes was analysed using multivariable modified Poisson regression with robust errors.^18^ Seven separate multivariable modified Poisson regressions were performed for the association between gender minority status and each binary outcome (model 1). Next, we used linear regression to estimate associations between gender minority status and the three continuous measures of reported psychological distress, behavioural and emotional difficulties (adolescent and parent/carer report). We adjusted estimates for sexual identity (model 2). To explore potential mechanisms, we then added to model 2 the hypothetical mediating substance use variables (model 3) and victimisation variables (model 4). Results for the binary outcomes are presented as relative risks (RRs) and continuous outcomes as coefficients, both with 95% confidence intervals. To examine the influence of missing data we re-ran the analysis on a complete data sample. All analyses were performed in Stata version 17.0 (Stata Corp.).

## Results

Supplementary Fig. 1 (available at https://doi.org/10.1192/bjo.2023.534) shows how we derived the analytical sample. Of the 10 247 participants, 113 (1.1%) reported they were a gender minority. Table 1 shows the characteristics of young people according to gender minority status. Compared with their cisgender peers, young people who identified as a gender minority were more likely to be bisexual (33.0 *v*. 17.2%) or gay (28.5 *v*. 2.2%) or report an ‘other’ sexual identity (30.3% *v*. 1.1%), and to report all forms of victimisation, including sexual assault (12.2 *v*. 3.1%), an unwelcome sexual approach (31.0 *v*. 12.2%), and experience of physical violence (36.9 *v*. 17.3%), but they were less likely to identify as an ethnic minority (21.3 *v*. 12.2%). There were no other differences in participant characteristics according to gender minority status.

**Table 1:** Characteristics of young people by self-reported gender identity

	Gender identity, %^a^		
Characteristic	Cisgender (*n* = 10 134)	Gender minority (*n* = 113)	*P*-value^b^
Demographics			
Resident in a rented property	28.0	33.2	0.31
One resident parent/ carer in household	28.1	32.7	0.33
Parent/carer unemployed	24.3	21.1	0.48
Ethnic minority	21.1	12.2	0.03
Sexual identity^c^			
Heterosexual	79.5	8.2	
Bisexual	17.2	33.0	<0.001
Gay	2.2	28.5	<0.001
Other	1.1	30.3	<0.001
Substance use			
Lifetime smoking	41.4	35.5	0.23
Lifetime alcohol use	78.9	80.8	0.62
Lifetime drug use	28.9	32.9	0.36
Victimisation			
Insulted you, threatened or shouted at you in public	38.6	65.2	<0.001
Spread gossip, ignored, other emotional abuse	38.3	63.7	<0.001
Been physically violent towards you	17.3	36.9	<0.001
Hit or used a weapon against you	3.1	8.5	0.002
Stolen something from you	8.1	16.8	0.001
Harassed via mobile phone or email	14.7	29.8	<0.001
Sent pictures of you/ rumours	7.0	17.0	<0.001
Made an unwelcome sexual approach	12.2	31.0	<0.001
Assaulted you sexually	3.1	12.2	<0.001

a. All numbers estimated from imputed proportions.

b. Determined by Poisson regression.

c. Bisexual comprised bisexual and mainly heterosexual/straight respondents; gay comprised mainly gay or lesbian and completely gay or lesbian.

Gender minority young people were three times more likely than their cisgender peers to report a suicide attempt (RR 3.42; 95% CI 2.45–4.78) (Table 2). There was evidence of an association of gender minority status with reporting self-harm in the previous year including cutting or stabbing (RR 4.38; 95% CI 3.55–5.40), burning (RR 3.81; 95% CI 2.49–5.82), bruising or pinching (RR 3.69; 95% CI 3.07–4.44), taking an overdose (RR 5.25; 95% CI 3.35–8.23), pulling out hair (RR 3.51; 95% CI 2.52–4.88), and harm in other ways (RR 6.39; 95% CI 4.63–8.83), as well as with scores on the Kessler K6 screening scale (coefficient 5.81; 95% CI 4.87–6.74) and SDQ total scores from study participants’ responses (coefficient 5.60; 95% CI 4.54–6.67) and parent/carers’ responses (coefficient 2.60; 95% CI 1.47–3.73). Associations were markedly reduced after the addition of sexual identity to models, but there was little evidence of further attenuation after substance use was added. After adjustment for reports of victimisation, the associations with gender minority status were weakened (Table 2).

**Table 2:** Self-reported suicide attempt, self-harm and psychological distress by gender minority status

			Relative risk (95% confidence interval)			
Outcome	Percentage		Model 1	Model 2	Model 3	Model 4
	Cisgender (*n* = 10 134)	Gender minority (*n* = 113)				
Suicide attempt	7.4	25.3	3.42 (2.45, 4.78)	1.77 (1.22, 2.57)	1.82 (1.28, 2.59)	1.19 (0.85, 1.66)
Self-harm						
Cut	10.8	47.3	4.38 (3.55, 5.40)	1.99 (1.58, 2.50)	2.02 (1.61, 2.53)	1.41 (1.12, 1.77)
Burned	4.5	17.3	3.81 (2.49, 5.82)	1.69 (1.06, 2.71)	1.77 (1.12, 2.79)	1.12 (0.72, 1.76)
Bruised or pinched	14.7	54.4	3.69 (3.07, 4.44)	1.77 (1.46, 2.15)	1.77 (1.46, 2.14)	1.35 (1.12, 1.62)
Overdose	3.0	15.8	5.25 (3.35, 8.23)	2.31 (1.36, 3.92)	2.38 (1.45, 3.92)	1.43 (0.87, 2.34)
Pulled hair	7.3	25.8	3.51 (2.52, 4.88)	1.58 (1.11, 2.25)	1.61 (1.14, 2.28)	1.10 (0.78, 1.56)
Other	4.3	27.5	6.39 (4.63, 8.83)	2.65 (1.82, 3.84)	2.59 (1.78, 3.79)	1.84 (1.26, 2.69)
Psychological distress	Mean (s.d.)		Coefficient (95% confidence interval)			
Kessler K6 screening scale total	7.21 (4.93)	13.02 (5.17)	5.81 (4.87, 6.74)	3.19 (2.24, 4.13)	3.23 (2.30, 4.16)	2.23 (1.37, 3.10)
Strengths and Difficulties (child)	11.23 (5.64)	16.83 (6.54)	5.60 (4.54, 6.67)	2.82 (1.72, 3.91)	2.88 (1.80, 3.95)	1.73 (0.71, 2.76)
Strengths and Difficulties (parent)	7.38 (6.04)	9.98 (6.87)	2.60 (1.47, 3.73)	0.73 (-0.46, 1.92)	0.83 (-0.36, 2.01)	0.21 (-0.96, 1.38)

Model 1: gender identity; Model 2: model 1 plus sexual identity; Model 3: model 2 plus substance use; Model 4: model 2 plus victimisation.

In the subset with no missing data, the confidence intervals for estimates overlapped with those from the main results using imputed data (Supplementary Table 1).

## Discussion

### Main findings

Gender minority adolescents were more likely to report ever making a suicide attempt, self-harm in the previous year, psychological distress, and behavioural and emotional difficulties than their cisgender peers. These associations were markedly reduced after accounting for sexual identity and reports of victimisation, but adjustment for substance use had little impact on the strength on associations. To our knowledge, this is the first study to provide nationally generalisable estimates of inequities in UK adolescents’ mental health according to gender identity and indicate that these differences may be related to exposure to victimisation.

### Interpretation of our findings and comparison with existing literature

The prevalence of young people identifying as a gender minority was small (1.2%) and comparable with estimates from community samples of young people in North America (2.1%, *n* = 65 231;^19^ 1.9%, *n* = 908)^20^ and the findings of the Youth’12 study, the only other nationally representative sample of high-school students conducted in New Zealand (1.2% transgender, *n* = 8,166).^6^ In agreement with the results of that study,^6^ we found gender minorities were around three times more likely to report having made a suicide attempt and were three to six times more likely to have self-harmed in the previous year than those who identified as cisgender. In two US studies, the online US Teen Health and Technology Study^4^ and The Youth Risk Behavior Survey^7^ conducted in ten US states, bullying and victimisation were reported more by transgender than cisgender young people. We replicated the findings of inequalities in mental health and victimisation according to gender identity but explicitly investigated whether victimisation explained associations between gender identity and mental health. Our analysis also extends the results of other studies by assessing six types of self-harm and a continuum of victimisation covering experiences ranging from insults to sexual assaults.

Among the mechanisms linking gender minority status with mental health problems, victimisation is likely to form part of an indirect mechanism. The marked attenuation of the association between gender minority status and outcomes we observed after adjustment for victimisation is consistent with it acting as a mediator. This hypothesis is consistent with the predictions of minority stress theory that mental health problems are more likely in gender minority compared with cisgender youth owing to the added stressors that accompany membership of this stigmatised group.^21,22^ Other studies with cisgender comparator groups have found that gender minority adolescents report more victimisation than their cisgender peers,^6,7^ providing support for this hypothesis. In contrast to previous studies,^4,7^ we found little difference in substance use by gender minority status. If these substances were being used to cope with victimisation, they may be better characterised as a downstream outcome of victimisation than a mediator of the association between gender identity and mental health problems.

### Limitations and strengths

One limitation of the present study is that its use of a single combined gender minority group meant that we did not further disaggregate analyses by gender identity (e.g. transmasculine, transfeminine, androgenous, gender fluid or non-binary) and did not include a ‘not sure about gender’ category. A related limitation is that we did not have enough young people to model all combinations of gender and sexual identity. There is likely to be variability in the lived experiences of different gender and sexual minority groups, and larger studies should investigate these differences. Some misclassifications could also have occurred if gender minority identities were underreported because of perceived stigma. This would lead to a misclassification which would probably attenuate associations to the null rather than introduce a spurious effect. Attrition and missing data are a concern in birth cohorts and can introduce selection biases. We used multiple imputation to maximise the plausibility of the missing-at-random assumption. Results were comparable when using the data-sets with no missing and imputed data. Our assessment of suicide attempts was a lifetime measure and so may reflect events that occurred before young people became aware of or identified as a gender minority. However, reverse cause, where a suicide attempt leads to a change in identity, seems a less plausible explanation for the associations reported than gender minority status acting as a putative causal factor.

The main strength of our study lies in the use of a large, contemporary, nationally representative sample of adolescents. Our findings are therefore likely to be generalisable across the UK. The use of a birth cohort with sex recorded by parents at a young age meant we doubled the number of people identified as a gender minority through a comparison of parent/carer reports of gender identity as a child with adolescent reports. This probably increased the power of the analysis and the precision of our estimates. Another strength was the assessment of self-harm that occurred in the past year, which is more clinically relevant and less prone to recall bias than assessments of self-harm occurring more than a year ago.^12^

### Implications

In conclusion, we found that gender minority adolescents were more likely to report symptoms of psychological distress, emotional and behavioural difficulties, and self-harm and to have made a suicide attempt than their cisgender peers. We extend the findings from previous studies by showing that adjusting for victimisation explains variation in the association between gender minority status and outcomes. The implication of this finding is that reducing victimisation may be helpful in narrowing the gap in mental health problems between gender minority and cisgender adolescents. The unquestioning acceptance of rigid concepts of gendered behaviour should be challenged by wider society, including young people's families and communities. Clinicians need to consider discussing self-harm and suicide with gender minority young people and help them find safer ways of coping. Policies, organisational practices, and school-based interventions should seek to reduce victimisation of gender minority young people.

## Data Availability

The MCS data are available to all researchers, free of cost, from the UK Data Service (https://www.ukdataservice.ac.uk).

## Supplementary material

For supplementary material accompanying this paper visit http://doi.org/10.1192/bjo.2023.534. click here to view supplementary material
