# Supplementary material for: Psychological distress, self-harm and suicide attempts in gender minority compared with cisgender adolescents in the UK
Source: BJPsych Open. 2023 Aug 1;9(5):e138. doi: 10.1192/bjo.2023.534 (PMC10486222; doi:10.1192/bjo.2023.534)
Supplement: Supplementary file 1 [file S2056472423005343sup001.docx]

**Data supplement**

1. eFigure 1. Flow diagram of participants included in the analytical sample
2. Table S1. Self-Reported Suicide Attempt, Self-Harm, and Psychological Distress by Gender Minority Status in a Sample with Complete Data (n = 7,829)

2000/2 recruited at birth (n = 19519)

Lost by 17-year follow-up

(n = 5023)

Invited to sweep at 17 years of age (n = 14496)

Did not respond

(n = 4151)

Children interviewed at 17 years of age (n = 10345)

Excluded as either did not know, preferred not to say, or did not want to provide gender, sexual or ethnic identity (n = 98)

Analytical sample (10247)

**eFigure 1. Flow diagram of participants included in the analytical sample**

**Table S1. Self-Reported Suicide Attempt, Self-Harm, and Psychological Distress by Gender Minority Status in a Sample with Complete Data (n = 7 829)**

|  | **Risk ratio (95% confidence interval)** | | | | | |
| --- | --- | --- | --- | --- | --- | --- |
| **Outcome** | **Model 1** | **Model 2** | **Model 3** | **Model 4** |  | |
|  |  |  |  |  | | |
| Suicide attempt | 3·28 (2·05, 5·23) | 1·68 (1·00, 2·81) | 1·80 (1·07, 3·02) | 1·15 (0·74, 1·79) | | |
| Self-harm |  |  |  |  | | |
| Cut | 5·29 (4·18, 6·69) | 2·40 (1·83, 3·14) | 2·50 (1·93, 3·23) | 1·69 (1·30, 2·19) | | |
| Burned | 5·06 (3·09, 8·29) | 2·19 (1·23, 3·89) | 2·39 (1·36, 4·20) | 1·48 (0·86, 2·54) | | |
| Bruised or pinched | 4·07 (3·32, 4·98) | 1·97 (1·58, 2·45) | 1·98 (1·59, 2·45) | 1·50 (1·22, 1·85) | | |
| Overdose | 5·57 (2·94, 10·54) | 2·82 (1·37, 5·82) | 3·14 (1·57, 6·28) | 1·85 (0·95, 3·62) | | |
| Pull hair | 3·99 (2·70, 5·91) | 1·82 (1·18, 2·80) | 1·89 (1·22, 2·90) | 1·24 (0·82, 1·88) | | |
| Other way | 6·86 (4·77, 9·89) | 2·77 (1·81, 4·25) | 2·72 (1·76, 4·22) | 1·97 (1·30, 2·99) | | |
|  |  |  |  |  | |  |
| **Psychological distress** | **Coefficient (95% confidence interval)** | | | | | |
| Kessler K6 screening scale total | 5·90 (4·89, 6·91) | 3·42 (2·40, 4·44) | 3·49 (2·48, 4·51) | 2·52 (1·57, 3·47) | | |
| Strengths and Difficulties total (child) | 5·31 (4·15, 6·47) | 2·51 (1·31, 3·70) | 2·61 (1·42, 3·80) | 1·50 (0·38, 2·62) | | |
| Strengths and Difficulties total (parent) | 2·22 (1·03, 3·41) | 0·42 (-0·84, 1·67) | 0·58 (-0·66, 1·83) | 0·05 (-1·18, 1·29) | | |

Model 1: gender identity; Model 2: model 1 plus sexual identity; Model 3: model 2 plus substance use; Model 4: model 2 plus victimisation
